# Cerebral schistosomiasis in a 3-year-old girl due to *Schistosoma japonicum*: a case report

**DOI:** 10.3389/fimmu.2024.1502627

**Published:** 2024-12-04

**Authors:** Yangyang Guo, Jindong Zhang, Ruichao Chai, Yanlin Yang, Kaibing Tian, Guangzhi Shi, Linlin Zhang

**Affiliations:** ^1^ Department of Critical Care Medicine, Beijing Tiantan Hospital, Capital Medical University, Beijing, China; ^2^ Department of Gastroenterology, Peking University Third Hospital, Beijing, China; ^3^ Department of Molecular Neuropathology, Beijing Neurosurgical Institute, Beijing Tiantan Hospital, Capital Medical University, Beijing, China; ^4^ Department of Neurosurgery, Beijing Tiantan Hospital, Capital Medical University, Beijing, China

**Keywords:** Cerebral schistosomiasis, pediatric, diagnosis, neurosurgery, case report

## Abstract

**Introduction:**

Cerebral schistosomiasis is a rare but severe manifestation of *Schistosoma japonicum* infection, often leading to significant neurological impairment. This case report details the clinical presentation, diagnostic challenges, and treatment of a 3-year-old girl with cerebral schistosomiasis in Sichuan, China.

**Case description:**

A 3-year-old girl from a rural area in Sichuan, China, presented with a 3-month history of unstable walking, left facial paralysis, drowsiness, and intermittent fever. Brain MRI revealed giant polycystic lesions in the right temporal, parietal, and occipital lobes, suggestive of an abscess with ependymitis. Despite no history of travel to endemic areas or known freshwater exposure, the patient was diagnosed with cerebral schistosomiasis due to *Schistosoma japonicum* based on histological examination and metagenomic next-generation sequencing (mNGS) of brain tissue obtained through surgery. The patient underwent surgical resection of the lesions and received two courses of praziquantel combined with corticosteroids and anticonvulsants. Despite residual left-sided hemiplegia, her cognitive function remained comparable to that of her peers, and no recurrence of the disease was observed over three years of follow-up.

**Conclusion:**

This case underscores the diagnostic challenges of cerebral schistosomiasis, particularly in non-endemic areas or in the absence of a clear history of freshwater exposure. Early surgical intervention combined with praziquantel treatment can lead to favorable outcomes, even in severe cases with extensive brain involvement.

## 1 Introduction

Schistosomiasis is a major tropical disease caused by blood-dwelling helminths of the genus *Schistosoma*, affecting over 230 million individuals across 74 countries, with symptomatic cases exceeding 120 million worldwide (1, 2). Among the various clinical manifestations, neuroschistosomiasis is particularly severe. Despite its seriousness, the prevalence of neuro schistosomiasis among individuals infected with *Schistosoma* remains poorly understood. The current literature lacks reliable epidemiological studies that accurately assess the prevalence of this condition, highlighting the need for further research and more comprehensive epidemiological studies to better understand its prevalence and impact.

This neurological involvement can lead to significant and often irreversible disabilities (3, 4). The spectrum of neurological symptoms associated with neuroschistosomiasis includes headache, altered mental status, seizures, sensory disturbances, ataxia, visual impairment, speech difficulties, and other focal neurological deficits depending on the lesion’s location (5).

Five Schistosoma species are known to infect humans: *Schistosoma mansoni* (prevalent in Africa, the Middle East, the Caribbean, and South America), *Schistosoma haematobium* (endemic in Africa and the Eastern Mediterranean), *Schistosoma japonicum* (found primarily in China, Japan, and the Philippines), *Schistosoma mekongi* (endemic in Southeast Asia), and *Schistosoma intercalatum* (endemic in Central West Africa) (2, 6).Transmission typically occurs through contact with contaminated freshwater during activities such as swimming, bathing, fishing, farming, or washing clothes (4).

Although gastrointestinal schistosomiasis caused by *Schistosoma japonicum* is relatively common, cerebral involvement is rare. Moreover, cases of cerebral schistosomiasis japonica are almost invariably associated with hepatosplenomegaly (7). In this report, we present an unusual case of cerebral schistosomiasis japonica in a 3-year-old child, notable for the absence of extracranial involvement. Written informed consent for publication was obtained from the patient’s legal guardians.

## 2 Case presentation

A 3-year-old female from Sichuan, China, went to the clinic and complained of unstable walking for 3 months, left facial paralysis, drowsiness, and intermittent fever for more than 2 months. Magnetic resonance imaging (MRI) showed the giant polycystic space-occupying lesions and the imaging diagnosis considered an abscess with ependymitis. She was brought up in a small village in Sichuan province, and her parents made a living by running a pig farm and planting rice. She had no travel history to Poyang and Dongting Lakes or other endemic areas in China, and no specific history of freshwater exposure.

**Figure 1:**
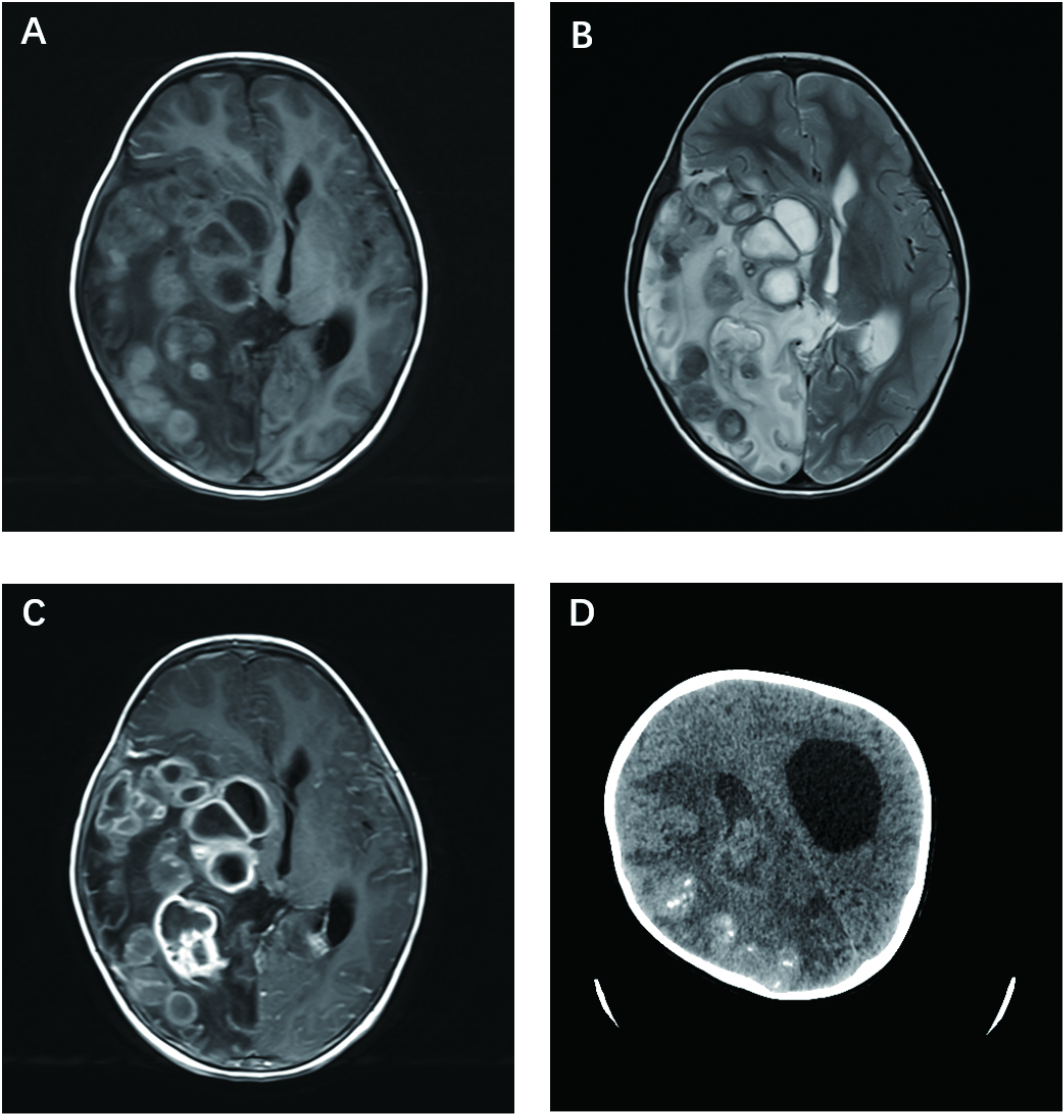
Cerebral schistosomiasis. **(A)** Axial T1-weighted brain MRI demonstrates mild hyperintense or hypointense cystic lesions in the right temporal, parietal, and occipital lobes. **(B)** Axial T2-weighted brain MRI shows mild hypointense or hyperintense cystic lesions in the right temporal, parietal, and occipital lobes, with surrounding patchy hyperintense signals indicative of edema. **(C)** Axial T1-weighted post-contrast MRI reveals moderate to marked enhancement in the cyst walls consistent with *Schistosoma japonicum* eggs. **(D)** Non-enhanced axial head CT scan shows irregular mixed density lesions in the right cerebral hemisphere and right lateral ventricle, with multiple calcifications and cystic densities, surrounded by edema.

The brain MRI revealed giant polycystic lesions in the right temporal, parietal, and occipital lobes (**Figures 1A–C**). These findings were corroborated by cranial computed tomography (CT), which also showed calcifications (**Figure 1D**).

No abnormality was found on her chest radiograph, abdominal ultrasound, and urinary ultrasound. A complete blood cell count showed mild eosinophilia (0.53 × 10^9^/L). Inflammatory markers, serum electrolyte levels, and liver function test results were within their respective reference range. No parasitic eggs were detected in urine or feces.

**Figure 2:**
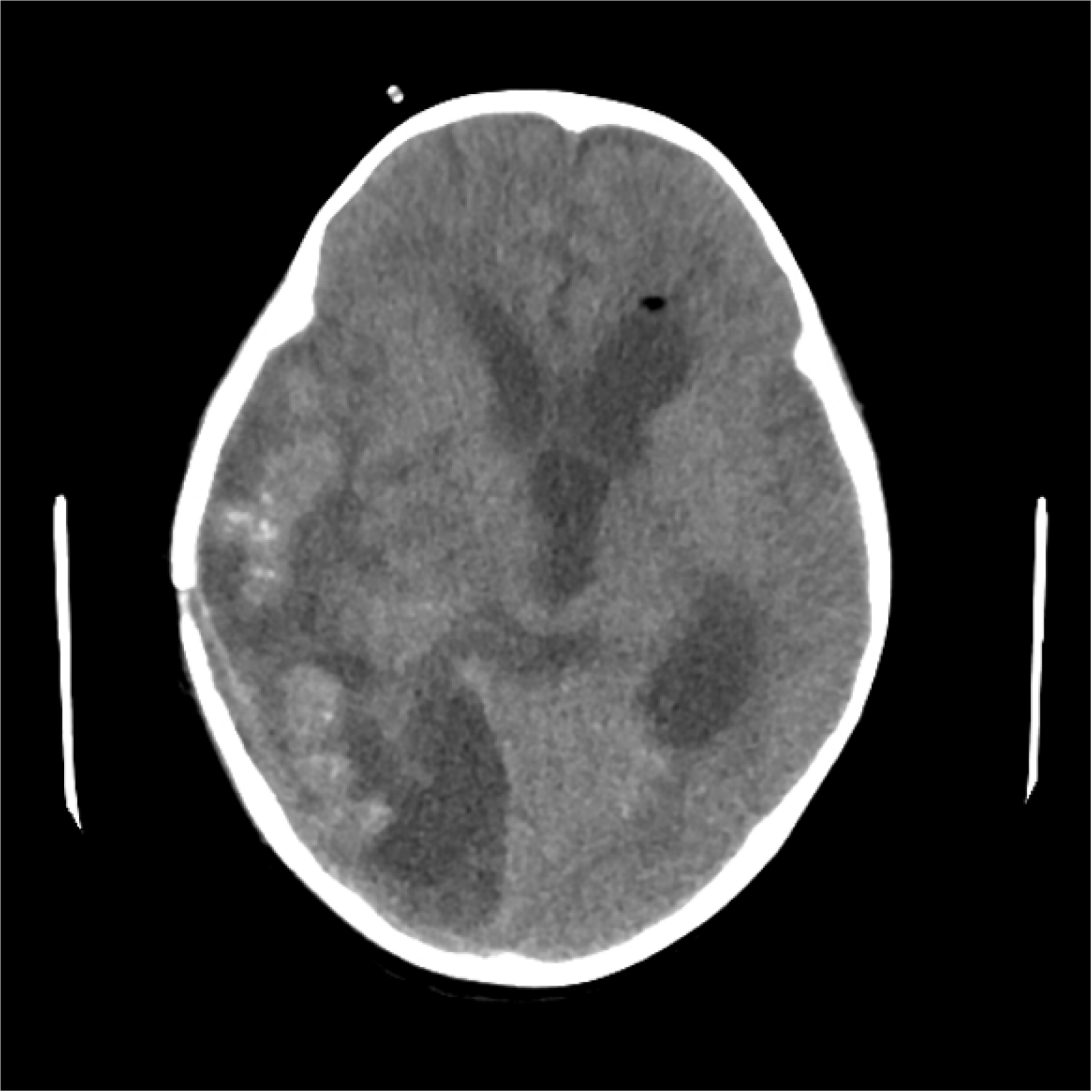
Postoperative cranial CT scan reveals a large mixed-density area in the right temporoparieto-occipital region, accompanied by a local surgical cavity and surrounding low-density areas.

**Figure 3:**
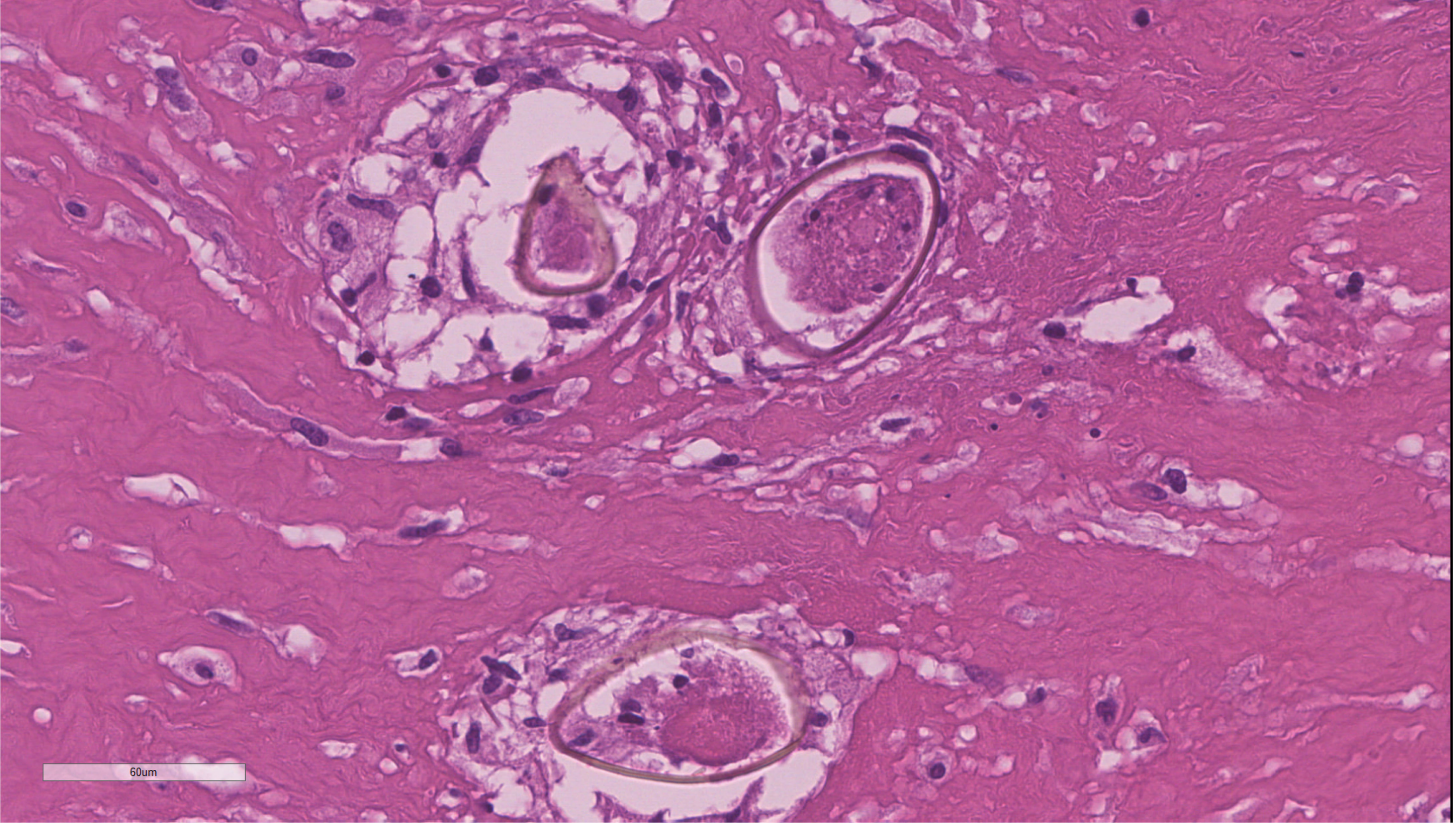
Histopathological examination of surgical specimens revealing brain parenchyma infiltrated by necrotizing granulomas containing *Schistosoma* eggs.

The patient underwent ventricle puncture for external CSF drainage and right parietal-occipital lobectomy for diagnosing and debulking of the lesion, and was transferred to the ICU for further monitoring and treatment post-operation. The postoperative cranial CT scan shows a large mixed-density area in the right temporoparieto-occipital region, with a local surgical cavity and surrounding low-density areas (**Figure 2**). CSF analysis revealed 205 leucocytes/uL, protein level increased to 740.4mg/dL and glucose level was within the reference range. The results of Gram staining and prolonged cultures were negative. Histological examination revealed parasitic granuloma in brain parenchyma with extensive multifocal necrosis, surrounded by fibrous tissue hyperplasia and inflammatory cell infiltration (**Figure 3**). The morphology of parasitic eggs was consistent with *Schistosoma* species, and the metagenomic Next-Generation Sequencing(mNGS)result of the pathological slices was confirmed as *Schistosoma japonicum*.

After the operation, abdominal and urinary ultrasounds were performed repeatedly, and no abnormalities were revealed. The result of repeat urine and feces testing for *Schistosoma* eggs was negative. Serum immunoglobulin G (IgG) enzyme-linked immunosorbent assays (ELISA) for *Schistosoma* species were negative in the patient and her family.

**Figure 4:**
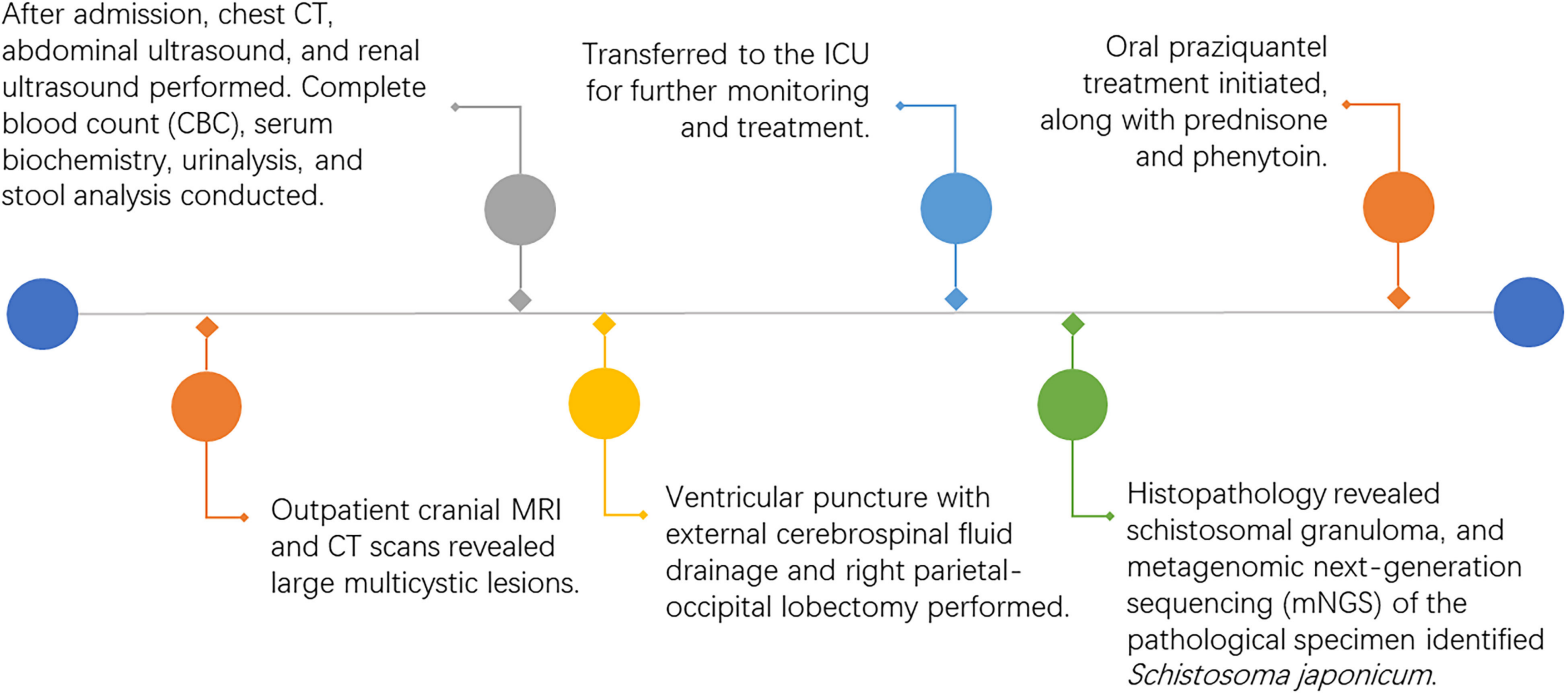
Timeline diagram of disease and treatment course.

The patients received two courses of oral praziquantel treatment, each lasting 10 days (40 mg/kg per day in 3 doses), at 6-week intervals, alongside oral prednisolone and phenytoin (**Figure 4**). Throughout the treatment, there were no adverse symptoms such as headache, nausea, or dizziness. Upon discharge, the patient was alert with left-sided hemiplegia and mild psychomotor slowing. Over the following three years, we conducted continuous follow-up and found no history of re-exposure. Additionally, annual serological tests consistently returned negative results, and follow-up head CT scans revealed no new lesions. Although the patient continued to experience left-sided hemiplegia, her cognitive function was comparable to that of her peers, and she achieved a Glasgow Outcome Scale (GOS) score of 4.

## 3 Discussion

Schistosomiasis is one of the most devastating tropical diseases, which is a helminthic infection caused by blood-dwelling flukes of the genus *Schistosoma (* 2, 8). More than 230 million people in 74 countries are affected by schistosomiasis worldwide and 120 million of them have symptoms (1, 2). Neuroschistosomiasis is one of the most severe clinical manifestations of the disease which can lead to profound and life-altering disability (3, 4). The five species that affect humans are *Schistosoma mansoni* (endemic in Africa, the Middle East, the Caribbean, and South America), *Schistosoma haematobium* (endemic in Africa and the Eastern Mediterranean), *Schistosoma japonicum* (endemic primarily in China, Japan, and the Philippines), *Schistosoma mekongi* (endemic in Southeast Asia), and *Schistosoma intercalatum* (endemic in Central West Africa) (2, 6). Infection is usually acquired through contact with contaminated freshwaters, such as swimming, bathing, fishing, farming, and washing clothes (4). Neuroschistosomiasis is almost entirely caused by *S. mansoni*, *S. haematobium*, or *S. japonicum*. Cerebral involvement tends to be more common in *S. japonicum*, and it is believed that the small size and rounded shape of the eggs enable them to migrate easily to the brain (5). Meanwhile, spinal cord involvement is more common in *S. mansoni* and *S. haematobium (* 6).

Cerebral schistosomiasis can be manifested as acute schistosomal encephalopathy and pseudotumoral encephalic schistosomiasis (PES) (6). Acute schistosomal encephalopathy presents with headache, seizures, blurred vision, visual field impairment, and speech disturbances. The pseudotumoral form of cerebral schistosomiasis is caused by tumor-like lesions surrounded by edema, the lesions in the brain causing increased intracranial pressure and focal neurological deficit (9). According to literature reports, PES often presents as one lesion, with few case descriptions of two or more discrete lesions (3). The multiple giant cystic space-occupying lesions described in our case have not been reported in the literature.

Schistosome infection is typically acquired through contact with contaminated freshwater, with the main transmission sites in China being Poyang and Dongting Lakes. The patient had no travel history to these areas. Although her family owns a pig farm and cultivates rice, she has never been in contact with pigs or visited the fields. We traced the infection history of her family, but no one else was infected, and serum IgG assays for Schistosoma species were negative in her family. Her brother, who is three years older than her, is also in good health.

Although paragonimiasis can cause cystic lesions in the CNS, we also conducted serum IgG assays for Paragonimus in the patient, which returned negative results. Furthermore, histopathological examination of the brain lesions revealed parasite eggs resembling those of Schistosoma japonicum. Additionally, metagenomic next-generation sequencing (mNGS) of the pathological samples confirmed the presence of Schistosoma japonicum. Therefore, we conclude that the diagnosis of schistosomiasis is clearly established in this case. Given these findings, we can only speculate that the patient may have had incidental exposure to infected freshwater in the past.

Cerebral schistosomiasis is not commonly encountered and is challenging to diagnose (10, 11). Magnetic resonance imaging (MRI) is important in diagnosis and differential diagnosis (12, 13). Like other organs, the granulomatous reaction in the brain is divided into three stages: necrotic-exudative stage, productive stage, and fibrosis healing stage. The MRI findings are closely correlated with these stages. Lu et al. (13) found that larger granulomatous lesions were hyperintense on T2W imaging and hypointense on T1W imaging, corresponding to histological necrosis, while smaller lesions were isointense or hypointense on T2W imaging and isointense on T1W imaging, corresponding to productive changes.

Neuroschistosomiasis is challenging to be diagnosed by laboratory examination. Eosinophilia in the blood can be detected, but may not be detectable at the onset (6). cerebrospinal fluid (CSF) examination can be routine or show non-specific findings that tend to show an inflammatory phenotype with elevated protein and leukocyte counts. Elevated eosinophils can be detected in CSF in 40% to 50% of cases (6). Although about 40% of patients with acute neuroschistosomiasis can detect Schistosoma eggs in feces, urine, and/or rectal mucosa (rectal biopsy), the detection of Schistosoma eggs in the above samples is not an essential requirement for diagnosis (14). de Wilton et al. (4) discovered that conducting serological tests for schistosomiasis on both serum and cerebrospinal fluid (CSF) in patients with a history of exposure to schistosomiasis-endemic areas and presenting with neurological symptoms is essential for improving diagnostic accuracy. However, a negative result on serological antibody detection does not rule out schistosomiasis (1). In this case, the absence of detectable *Schistosoma japonicum* eggs in the feces, along with a lack of evidence for hepatic or intestinal infection, suggests that the worms may have migrated to ectopic sites within the venous plexus or intracranial veins, directly depositing eggs into the central nervous system.

Because the neuroimaging and laboratory examinations are non-specific, the definitive diagnosis can only be made by finding evidence of Schistosoma eggs and granulomatous response through pathological examination (5). Biopsy of the brain may show Schistosoma eggs in various stages of evolution, with surrounding inflammatory reactions. The granuloma around the eggs has a necrotic center that contains *schistosome* egg and/or an egg cluster surrounded by epithelioid cells, giant cells, and lymphocytes, as well as an outer layer of eosinophils, fibroblasts, and plasma cells (6).

Praziquantel is the anti-parasitic agent of choice for all *schistosome* species with a reported cure rate of 70–90% (5, 6). This drug provokes the disruption of parasite tegument, depletes the glutathione content, and affects the function of the calcium channel. Adverse reactions are generally mild, including dizziness, headache, fatigue, abdominal pain, and lower limbs pain (15). Although there is no clear consensus on the treatment time for neuroschistosomiasis, the recommended course of treatment is 1 day to 2 weeks (16). Dosing regimens vary from 40–60 mg/kg/day, and they are given in divided doses to treat neuroschistosomiasis (4). Surgical treatment is usually indicated for patients with enlarged intracranial lesions with mass effect, poor response to praziquantel, inflammatory encephaledema, obstruction of CSF circulation, cerebral hernia, and exclusion of gliomas and other brain diseases (12).

This case report provides valuable insights into a rare instance of cerebral schistosomiasis japonica in a child without extracranial involvement. The strengths include a thorough diagnostic approach, using advanced imaging, histopathology, and mNGS, which confirmed the diagnosis despite negative serological tests. However, the lack of a clear exposure history and negative serological tests in the patient and her family limit the understanding of transmission and diagnostic sensitivity. The persistence of neurological deficits also underscores the need for improved treatment strategies.

Overall, this case report adds to the growing body of literature on neuroschistosomiasis, particularly in pediatric patients, while also highlighting the need for further research into the diagnostic and therapeutic challenges associated with this rare manifestation.

## Data Availability

The original contributions presented in the study are included in the article/supplementary material. Further inquiries can be directed to the corresponding authors.
